# Body Characteristics, Dietary Protein and Body Weight Regulation. Reconciling Conflicting Results from Intervention and Observational Studies?

**DOI:** 10.1371/journal.pone.0101134

**Published:** 2014-07-03

**Authors:** Mikkel Z. Ankarfeldt, Lars Ängquist, Tanja Stocks, Marianne U. Jakobsen, Kim Overvad, Jytte Halkjær, Wim H. M. Saris, Arne Astrup, Thorkild I. A. Sørensen

**Affiliations:** 1 Institute of Preventive Medicine, Bispebjerg and Frederiksberg Hospital, The Capital Region, Copenhagen, Denmark; 2 Faculty of Medical and Health Sciences, University of Copenhagen, Copenhagen, Denmark; 3 Department of Clinical Sciences in Malmö, Diabetes and Cardiovascular Diseases, Genetic Epidemiology, Lund University, Lund, Sweden; 4 Department of Surgical and Perioperative Sciences, Urology and Andrology, Umeå University, Umeå, Sweden; 5 Section for Epidemiology, Department of Public Health, Aarhus University, Aarhus, Denmark; 6 Danish Cancer Society, Research Center, Copenhagen, Denmark; 7 NUTRIM School for Nutrition, Toxicology and Metabolism, University of Maastricht, Maastricht, the Netherlands; 8 Department of Nutrition, Exercise and Sports, NEXS, Faculty of Science, University of Copenhagen, Frederiksberg, Denmark; 9 The Novo Nordisk Foundation Centre for Basic Metabolic Research, Faculty of Medical and Health Sciences, University of Copenhagen, Copenhagen, Denmark; National Institute of Agronomic Research, France

## Abstract

**Background/Objectives:**

Physiological evidence indicates that high-protein diets reduce caloric intake and increase thermogenic response, which may prevent weight gain and regain after weight loss. Clinical trials have shown such effects, whereas observational cohort studies suggest an association between greater protein intake and weight gain. In both types of studies the results are based on average weight changes, and show considerable diversity in both directions. This study investigates whether the discrepancy in the evidence could be due to recruitment of overweight and obese individuals into clinical trials.

**Subjects/Methods:**

Data were available from the European Diet, Obesity and Genes (DiOGenes) post-weight-loss weight-maintenance trial and the Danish Diet, Cancer and Health (DCH) cohort. Participants of the DCH cohort were matched with participants from the DiOGenes trial on gender, diet, and body characteristics. Different subsets of the DCH-participants, comparable with the trial participants, were analyzed for weight maintenance according to the randomization status (high or low protein) of the matched trial participants.

**Results:**

Trial participants were generally heavier, had larger waist circumference and larger fat mass than the participants in the entire DCH cohort. A better weight maintenance in the high-protein group compared to the low protein group was observed in the subgroups of the DCH cohort matching body characteristics of the trial participants.

**Conclusion:**

This modified observational study, minimized the differences between the RCT and observational data with regard to dietary intake, participant characteristics and statistical analysis. Compared with low protein diet the high protein diet was associated with better weight maintenance when individuals with greater body mass index and waist circumference were analyzed. Selecting subsets of large-scale observational cohort studies with similar characteristics as participants in clinical trials may reconcile the otherwise conflicting results.

## Introduction

Physiological evidence indicates that a high intake of protein may increase thermogenic response and reduce caloric intake by increased satiety [1]–[3]. Randomized, controlled trials (RCTs) have suggested an overall beneficial effect of high-protein diets on weight loss and weight maintenance after weight loss [4], [5]. In contrast, large-scale, long-term observational cohort studies have shown that greater protein intake is associated with weight gain [6], [7]. Although results from RCTs and observational studies often reach similar results [8]–[12], the sometimes conflicting findings make the formation of health recommendations difficult. Hernán *et al.* [13] addressed the conflicting results for the association between hormone replacement therapy in postmenopausal women and risk of coronary heart disease. In an analysis where characteristics of the RCT was mimicked in the observational data, the association in the modified observational study approximated the result of the RCT. Potentially, other areas in medicine showing diverse results in observational studies versus RCTs may also be due to different participant and study characteristics rather than by diverse exposure-disease associations *per se*.

In the study of dietary protein and weight regulation, the results from RCTs and observational studies are based on average weight changes, and show considerable diversity in both directions. RCTs have commonly investigated overweight and obese individuals only, while observational studies have also included normal and underweight individuals. Moreover, the dietary protein intake in the high-protein arm of RCTs has been much higher than the average habitual protein intake in observational studies. We speculate if these differences are important to find an, on average, beneficial effect of protein.

This would correspond to effect-modification by the selection criteria. Individuals recruited for the trial could be represented by a subset of the broader population included in the observational study. Identifying such subset of individuals in observational data, and making the statistical analysis similar to the trial counterpart, may resolve what seemed to be conflicting results.

The aim of the present study was to investigate whether subgroups of participants from a cohort study comparable to participants from a trial experiencing a beneficial effect of dietary protein on weight maintenance could be identified.

## Subjects and Methods

The participants in the DiOGenes trial [5], showing better weight loss maintenance with a high protein intake, were matched on gender, diet and body characteristics in the observational Danish Diet, Cancer and Health (DCH) cohort study, showing a tendency to weight gain with greater protein intake [6].

The DiOGenes trial [5] had an initial eight weeks low-calorie diet (LCD) weight loss phase, and investigated how the ratio of protein-carbohydrate intake and glycemic index (GI) influenced weight maintenance during six months (mean durations). The participants were overweight or obese adults from eight European countries. Out of 773 participants completing the weight loss phase, 548 completed the weight-maintenance intervention of one of five randomly allocated, ad libitum diets, all low in fat (25–30 energy percent [E%] fat). The five intervention diets were: 1) low protein (13 E%) and low GI, 2) low protein and high GI, 3) high protein (25 E%) and low GI, 4) high protein and high GI, or 5) a control diet based on local recommendations of a healthy diet. Weight was measured at randomization and during the intervention period by trained personnel. Three-day food diaries were obtained approximately four weeks after randomization. The trial has been described in detail elsewhere [5] and is registered with ClinicalTrials.gov, number NCT00390637.

**Figure 1:**
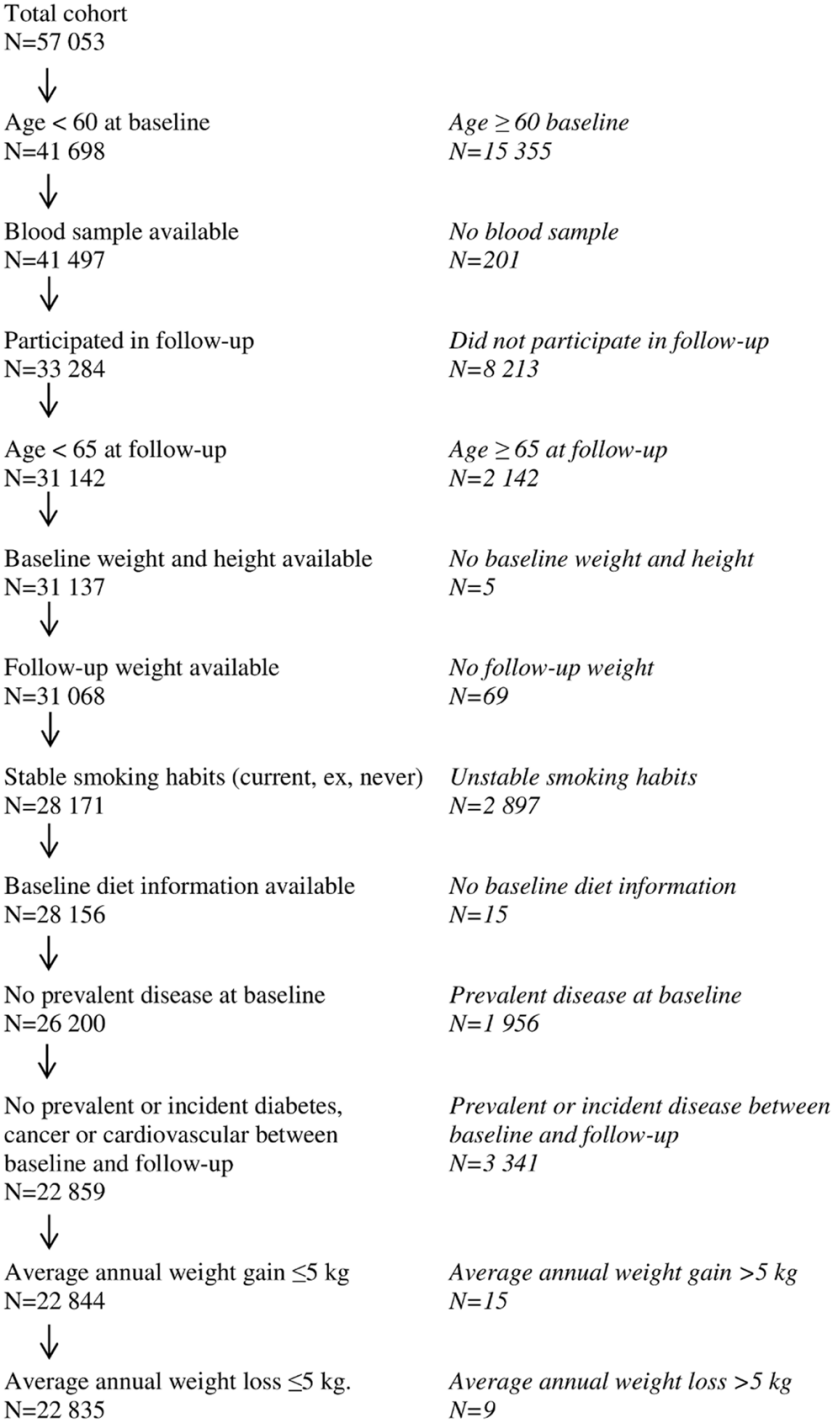
Flow chart of the Danish Diet, Cancer and Health cohort.

In the DCH cohort study, individuals living in the area of Copenhagen or Aarhus, Denmark, aged 50–64 without a diagnosis of cancer registered in the Danish Cancer Registry were invited. Baseline diet was obtained by a validated food frequency questionnaire. Weight was measured by trained personnel at baseline and obtained by self-measurements at follow-up five years later (mean duration). The DCH cohort study has been described in detail elsewhere [14]. Out of 160 725 invited, 57 053 individuals were examined. In accordance with the observational DiOGenes study [15], a generally healthy sub-cohort with available information was selected for further analyses. The following inclusion criteria were employed: available measures on weight at baseline and at follow-up, available measure of baseline height, available measures of dietary intake, stable smoking habits, available blood sample, age at baseline <60 years and age at follow-up<65 years, average weight gain ≤5 kg/year, absence of known diabetes, cancer or cardiovascular disease diagnosed before or during the follow-up period. As an indicator of health status, individuals with a weight loss >5 kg/year were also excluded. In total, 22 835 individuals, ranging from underweight to obese, met these criteria, and thus constituted the final study population for the present study (Figure 1). When investigated as a part of the DiOGenes observational study [6], the DCH cohort showed a tendency towards an association between greater protein and weight gain when analyzed with an energy partition model in a multiple linear regression, but without statistical significance.

Access to the data from both studies; the DiOGenes trial data and the DCH cohort data have been generated for many other purposes than the present study. Access to the data requires an application submitted to and subsequently approved by the respective Steering Boards of the studies. Contact professor Wim HM Saris (W.Saris@maastrichtuniversity.nl) and head of research Anne Tjønneland (annet@cancer.dk) for request to acces to the DiOGenes trial data and to the Danish Diet, Cancer and Health cohort data, respectively. Participants in both the DiOGenes trial and the DCH cohort provided written informed consent, and the studies were approved by the relevant scientific committees [14], [16].

### Matching

Matching was implemented in a sex-specific manner (i.e. women were matched with women, and men with men). The main matching variables were the dietary exposure variables used in the trial [5] (intake of protein E%, carbohydrate E% and GI) to aim for a similar distribution of the diet in the selected DCH participants as in the trial. As mentioned, only overweight or obese individuals were included in the DiOGenes trial, while body size was not an inclusion criterion in the DCH cohort study. Hence, body characteristic may potentially be effect-modifiers. The following variables describing body characteristics were identified for matching: body mass index (BMI, kg/m^2^), waist circumference (WC) and fat mass index (FMI; calculated by dividing kg of fat mass with height-squared in meters, kg/m^2^). In total, five combinations were matched: 1) only on dietary variables (protein E%, carbohydrate E% and GI), 2) dietary variables and BMI, 3) dietary variables and WC, 4) dietary variables and FMI or 5) dietary variables, BMI and WC.

The matching was based on similarities calculated by the *normalized Euclidean distance metric* [17] on the defined sets of variables. Trial participants were sequentially considered and the available DCH participant that, in each case, showed the closest match (the smallest distance) was selected without replacement. The normalization was made such that each contributing distance term was weighted by the inverse of the variance of the corresponding variable within the DCH cohort; thus, all variables were effectively treated as standardized to a unit standard deviation within the cohort.

Since matching was done without replacement and by sequentially scanning through the trial data, the matching could depend on the initial order of the individuals in the trial dataset. To take this into account, matching was done based on ten distinct random orders of the trial dataset.

**Figure 2:**
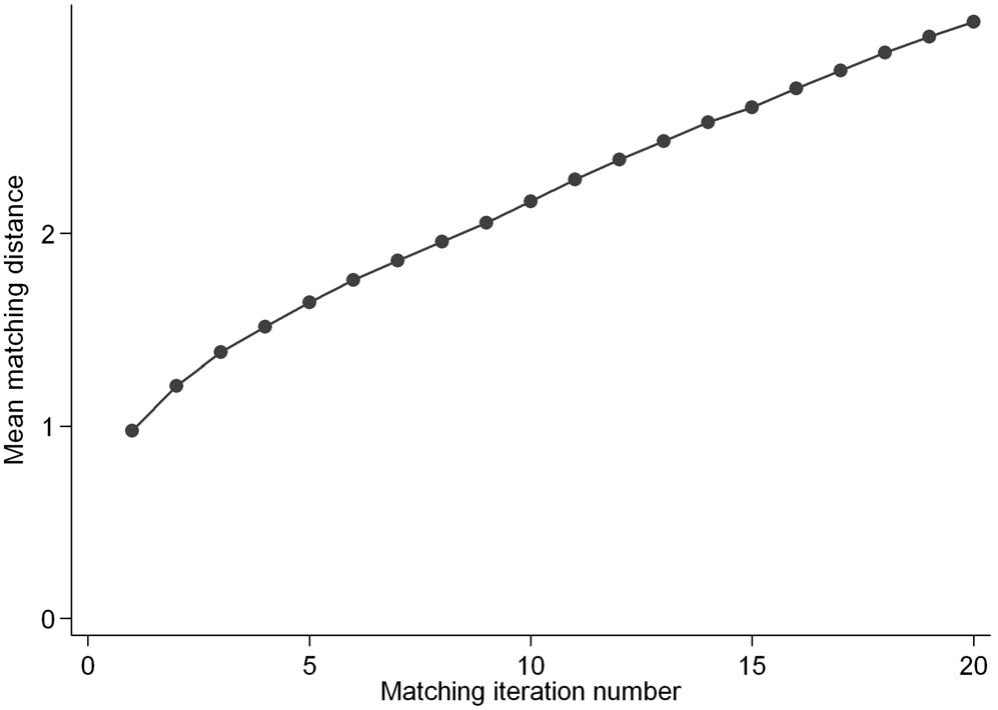
Scree plot. Mean matching distance across match-variables plotted against matching iteration number (number of observational participants matched with every trial participant). Matching was based on protein E%, carbohydrate E%, glycemic index, body mass index and waist circumference. Example of one of the ten random orders of the trial dataset is shown. E%: percent of energy intake.

To increase the sample size and hence statistical power, multiple DCH participants were matched to each trial participant. After the first full scan of the trial data, a second iteration was started, etc. However, with an increasing number of iterations, the distances of the matches increase; hence there is a trade-off between sample size and matching quality. To decide how many DCH participants to match each trial participant, scree plots (mean matching distance scores plotted against matching iteration numbers) [18] were inspected. Figure 2 shows, as an example, a scree plot, based on one of the random orders of the trial participants, when matching on protein E%, carbohydrate E%, GI, BMI and WC. Corresponding graphs based on other matching-combinations looked similar (not shown). After the third iteration, the distance-increase began to level off in all scree plots, so four iterations were used, i.e. four DCH participants were matched to each trial participant.

The group of individuals selected from the initial observational cohort data chosen by matching is referred to as the modified observational data below.

**Figure 3:**
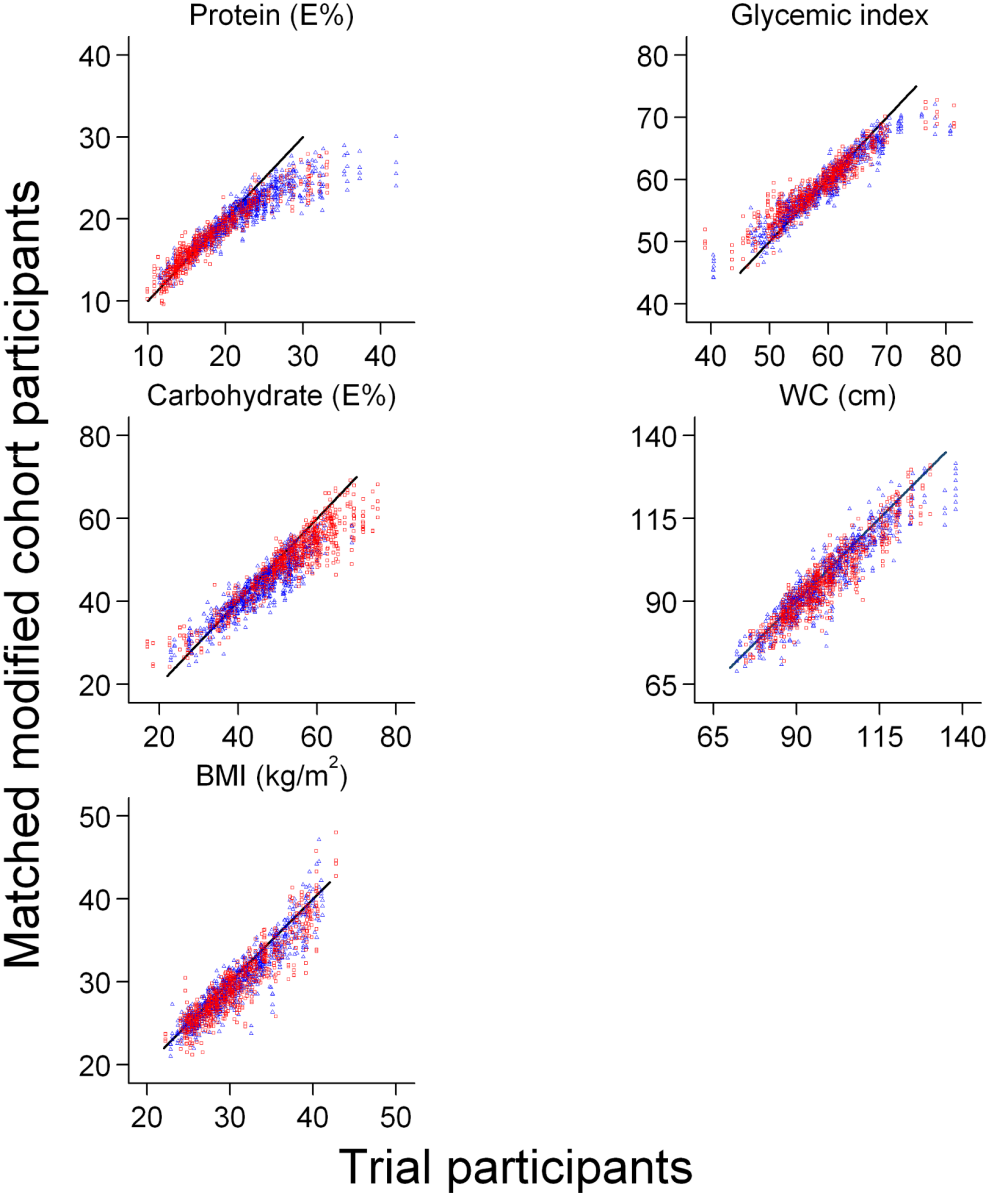
Matching performance. Scatter plot of the selected cohort participants vs. the corresponding, matched trial participant. Matching was based on protein E%, carbohydrate E%, glycemic index, BMI and WC. Four participants from the cohort data where matched with every trial participant. Mean values across matches of the ten random orders of the trial dataset are shown. The line of equality, y = x, indicates a perfect match. Red square marker: low protein group. Blue triangle marker: high protein group. Control group is not shown. Trial participants N = 428, defined by having available measurements of diet, BMI and WC; matched modified cohort participants N = 1 712. BMI: body mass index. E%: percent of energy intake. WC: waist circumference.

Figure 3 shows the matching-performance when matching on protein E%, carbohydrate E%, GI, BMI and WC, based on mean values across matches of the ten random orders of the trial dataset. For each match-variable, the values of the four DCH participants are plotted against the value of the matched trial participant. Corresponding plots based on other match-combinations looked similar; see Figures S1–S4 in file S1. A hypothetical, perfect match would have followed the straight line of equality (y = x). As seen, it was not possible to get a very close match in the observational data of the greatest protein E% intakes of the trial participants. A similar, although much less prominent, pattern was observed regarding match on carbohydrate E% and GI. The match on BMI and WC was fair, even though deviations tended to increase with higher values.

Figure 3 also distinguishes between the high and low protein group (using red square markers and blue triangle markers, respectively). Considerable variation in protein- and carbohydrate intake was present within the groups of trial participants randomized to high or low protein, and hence also among the participants of the modified observational data.

### Statistical analyses

Multiple linear regressions were used in the analyses of the modified observational data. In the trial [5] participants were analyzed according to randomization status in an intention-to-treat manner. To analyze the modified observational data in a similar manner, the selected DCH participants were analyzed according to the randomization status of the trial participant they matched. Similar to the analyses in the trial [5], the five groups (low protein, low GI; low protein, high GI; high protein, low GI; high protein, high GI; control) were recoded into three indicator variables: High protein (yes/no), high GI (yes/no) and control group (yes/no). This was the exposure in the modified observational data. Since dietary adherence was not taken into account in the analyses of the DiOGenes trial [5], it was not done in the analyses of the modified observational data.

The outcome in the DiOGenes trial [5] was weight change during the intervention. In the analyses of the modified observational data, the weight change from baseline to follow-up was used as the outcome. Since follow-up time varied within the DCH cohort, average annual weight change was calculated (kg/year).

Adjustment for potential confounding was implemented at two levels: 1) a model with adjustment for baseline BMI and gender similar to the analysis of the trial [5], and 2) a fully adjusted model with adjustment for gender (three groups: men, women without hormone use, women with hormone use), baseline BMI, age, physical activity (four groups: inactive, moderately inactive, moderately active, active), education (four groups: primary school, technical/professional school, secondary school, university degree) and baseline intake of fibers (grams/day) and alcohol E%. No adjustment was done for other macronutrients, since exposure status reflects dietary intake. Total energy intake was not adjusted for in the main analyses, in order not to adjust for a potential satiating effect of protein.

Regression analyses were performed for each match-combination and for each of the ten random orders of the trial dataset. Mean values of the ten estimated individual regression coefficients and standard errors of weight change were calculated and summary p-values derived and presented as the core results.

Statistical analyses were performed in Stata 12.1 (StataCorp LP, College Station, Texas).

### Supplementary analyses

First, analyses of the modified observational data were performed with additional adjustment for energy intake.

Second, since information on dietary intake was missing for some trial participants and therefore not matched to the DCH participants, the trial data were re-analyzed on the subset with dietary data available. This was performed as a linear regression based on weight change between randomization and post-intervention and included variables of assigned intervention diet, coded as indicator variables, gender and baseline BMI.

Third, because of the difficulties in matching on the highest protein intake reported by trial participants (Figure 3), the trial data were also re-analyzed similar to above, but with participants with a protein intake <30 E%.

## Results

Table 1 shows characteristics of trial participants, individuals in the initial DCH cohort and the modified observational cohort data when matching on protein E% and carbohydrate E%, GI, BMI and WC. Characteristics of the modified observational cohort data is shown as mean values across matches of the ten random orders of the trial dataset. Corresponding tables based on other match-combinations looked similar; see Table S1 in file S1. The initial cohort data showed a median protein intake comparable to the low protein group of the trial and a median intake of carbohydrate similar to the high protein group of the trial. Median intake of protein, carbohydrate and GI were similar in the high and low protein groups in the trial data and in the modified observational data. The ranges between the 5^th^ and 95^th^ percentiles of protein, carbohydrate and GI in the trial data and the modified observational data were all broad; however, the greatest variation was seen among the trial participants. The mean, 5^th^ and 95^th^ percentiles of body measures (BMI, WC and FMI) were slightly greater in the trial data than in the modified observational data. However, the mean, 5^th^ and 95^th^ percentiles of the body measures in the initial cohort was lower than the modified observational data, indicating that individuals in the modified observational data were more similar to the trial participants according to these variables.

**Table 1:** Baseline characteristics of the trial, initial cohort data and modified cohort data matched on protein E%, carbohydrate E%, glycemic index, BMI and WC^a.^

	DiOGenes trial		Initial DCH cohort	Modified DCH cohort data	
	N = 774		N = 57 053^b^	N = 2 180	
	*Low protein* c	*High protein* d		*Low protein* e	*High protein* f
	*N = 196*	*N = 232*		*N = 784*	*N = 928*
	*P50 (P5; P95)*	*P50 (P5; P95)*	*P50 (P5; P95)*	*P50 (P5; P95)*	*P50 (P5; P95)*
*Protein E%*	16.7 (12.3; 28.8)	21.4 (14.4; 31.2)	16.7 (12.9; 21.0)	17.1 (12.8; 24.3)	20.4 (15.0; 25.4)
*Carbohydrate E%*	53.9 (30.6; 68.4)	46.6 (32.2; 58.0)	44.2 (34.0; 55.4)	50.5 (32.5; 61.4)	44.3 (32.8; 54.4)
*Fat E%*	27.8 (15.6; 43.5)	29.9 (19.8; 47.1)	33.0 (23.7; 40.9)	29.2 (19.9; 39.1)	31.7 (23.6; 40.9)
*Alcohol E%*	0.0 (0.0; 7.0)	0.0 (0.0; 10.1)	4.2 (0.2; 18.6)	2.1 (0.1; 11.2)	2.7 (0.1; 15.8)
*Glycemic index*	59.1 (49.6; 67.8)	59.6 (49.5; 69.2)	60.0 (53.6; 66.5)	59.0 (51.2; 66.8)	58.9 (51.1; 67.8)
*Energy (MJ)*	5.8 (2.8; 10.1)	5.9 (3.5; 10.5)	9.5 (5.9; 14.7)	8.2 (4.9; 13.0)	7.9 (4.9; 12.6)
*Weight (kg)*	85.0 (67.0; 116.0)	87.4 (65.9; 116.4)	74.5 (54.9; 100.4)	82.0 (65.1; 110.3)	83.5 (65.2; 112.4)
*BMI (kg/m^2^)*	30.0 (24.8; 39.4)	29.9 (24.8; 38.9)	25.6 (20.4; 33.4)	28.9 (24.0; 38.3)	29.2 (24.2; 38.1)
*WC (cm)*	96.4 (80.9; 120.3)	95.8 (80.0; 120.3)	89.0 (69.0; 110.0)	94.0 (79.0; 118.0)	94.0 (78.0; 117.0)
*FMI (kg/m^2^)*	11.1 (6.0; 18.2)	10.3 (5.6; 18.7)	7.7 (4.2; 13.9)	10.6 (6.1; 17.6)	10.7 (5.8; 17.8)
*Age (years)*	41.7 (32.4; 51.6)	42.9 (31.5; 53.1)	56.0 (50.0; 64.0)	54.0 (50.0; 58.0)	53.0 (50.0; 58.0)
*Weight change (kg)* g	1.4 (−6.9; 7.4)	0.7 (−11.0; 7.1)	0.0 (−1.5; 1.3)	−0.1 (−1.7; 1.7)	−0.1 (−2.1; 1.5)

Abbreviations: BMI, body mass index; DCH, Diet Cancer and Health; DiOGenes, Diet Obesity and Genes; E %, percent of energy intake; FMI, fat mass index; P50, median; P5, 5^th^ percentile; P95, 95^th^ percentile; WC, waist circumference.

a Median, 5^th^ and 95^th^ percentile of baseline variables and weight change. Information on control groups is not shown. In trial data: high and low protein group is based on random allocation to intervention diet. In modified cohort data: high and low protein group is based on the randomization status of the matched trial participants. Mean values across matches of the ten random orders of the trial dataset are shown.

b Individuals with dietary information N = 56 998; weight N = 57 013; BMI N = 57 009; WC N = 57,000; FMI N = 56 906; age N = 57 053; weight change N = 43 661; glycemic index only available in the observational DiOGenes study-database N = 22 835.

c Individuals with information on fat mass (kg), FMI and body fat % N = 169; weight change N = 147.

d Individuals with information on fat mass (kg), FMI and body fat % N = 192; weight change N = 189.

e Individuals with information on fat mass, FMI and body fat % N = 782.

f Individuals with information on fat mass, FMI and body fat % N = 925.

g In trial data: change in weight during weight loss maintenance phase (mean 6 months). In observational data: average annual change in weight from baseline to follow-up (mean 5.3 years).

### Analyzing the modified observational data

Table 2 shows the results of the analyses of the modified observational dataset from the five distinct match-combinations.

**Table 2:** Annual weight change effect when comparing cohort individuals matching trial participants randomized to either high or low protein intake^a.^

Matching variables	Adjusted similar to trial^b^		Fully adjusted^c^	
	*β (range)*	*p-value* d *(range)*	*β (range)*	*p-value* d *(range)*
*Diet*	0.011 (−0.012; 0.034)	0.807 (0.422; 0.999)	−0.004 (−0.027; 0.020)	0.934 (0.543; 0.955)
*Diet and BMI*	−0.074 (−0.138; −0.031)	0.155 (0.008; 0.557)	−0.090 (−0.152; −0.049)	0.083 (0.003; 0.350)
*Diet and WC*	−0.063 (−0.103; −0.006)	0.208 (0.042; 0.903)	−0.090 (−0.131; −0.032)	0.076 (0.010; 0.530)
*Diet and FMI*	−0.074 (−0.125; −0.030)	0.192 (0.027; 0.594)	−0.079 (−0.132; −0.040)	0.166 (0.021; 0.482)
*Diet, BMI and WC*	−0.118 (−0.154; −0.051)	0.025 (0.004; 0.331)	−0.142 (−0.180; −0.078)	0.008 (<0.001; 0.142)

Abbreviations: BMI, body mass index; E%, percent of energy intake; FMI, fat mass index; WC, waist circumference.

a Five match-combinations: Diet only (Protein E%, carbohydrate E% and glycemic index), diet in combination with BMI, WC or FMI, or diet, BMI and WC. Multiple linear regression analysis was used. Exposure was indicator variables (yes/no) of matched randomization groups: high protein, high glycemic index, control. Outcome was average annual weight change between baseline and follow-up (mean 5.3 years). β = difference in body weight change (kg) between high and low protein group. β and p-values presented as means and summary statistics, respectively, complemented with corresponding ranges across matches of the ten random orders of the trial dataset.

b Adjustment for BMI and sex (male/female).

c Adjustment for sex (male/female without hormone use/female with hormone use), baseline BMI, age, physical activity (4 groups: inactive, moderately inactive, moderately active, active), education (4 groups: primary school, technical/professional school, secondary school, university degree) and intake of fibers (g/day) and alcohol (E%).

d Summary p-values, derived from the means of the β-estimates and of the corresponding standard errors, respectively, over the ten individual matches.

When matched on the dietary variables only, no difference on average annual weight change was seen between the high and low protein groups.

When matched on dietary variables in combination with BMI and WC, simultaneously, the high protein group had significantly lower weight gain, hence better weight maintenance, than the low protein group. The other three match-combinations with dietary variables and BMI, WC and FMI, respectively, showed the same tendency, although weaker and not reaching significant p-values.

Results from the two adjustment schemes were overall similar.

### Supplementary analyses

Comparing results based on fully-adjusted models with/without additional adjustments for total energy intake showed similar results (see Table S2 in file S1).

When analyzing trial participants with available information on diet and weight at randomization and post-intervention (N = 441), the high protein group had a better weight loss maintenance than the low protein group (adjusted mean difference: −1.20 kg, 95% CI: −2.35; −0.05, p = 0.041). When restricting trial participants further to those with a protein intake<30 E%, (N = 420), a similar result was obtained (−1.31 kg, 95% CI: −2.49; −0.13, p = 0.0301). These results are similar to the results reported in the initial trial [5], see Table S3 in file S1.

## Discussion

The physiological mechanism supposed to provide a beneficial effect on weight control of a high protein diet is believed to be universally valid. A beneficial effect is seen in RCTs among overweight or obese individuals [4], [5]. However the opposite is seen in observational studies investigating populations including also under- and normal-weight individuals [6], [7]. This study explored the possibilities of reconcile the conflicting evidence. Subgroups from the DCH cohort comparable to participants in the DiOGenes trial [5] were selected. Matching was based on to gender, macronutrient composition of the diet and body characteristics (BMI, WC or FMI, respectively, or BMI and WC). Weight change of the individuals matching the trial participants randomized to a high protein diet was compared to weight change of the individuals matching the trial participants randomized to a low protein diet. In these modified observational data, a lower weight gain, hence a better weight maintenance, was seen in the high-protein group than in the low-protein group. When matched only on diet there was no difference.

These findings suggest that the physiological mechanism behind a better weight control with a high protein intake should be reconsidered. A high intake of protein increases of satiety and thermogenesis [1]–[3]. Most of these studies were executed in overweight or obese subjects. From the results of the present study, it may be speculated that these beneficial effects are only present given a certain level of adiposity. If this is the case the effects on satiation and thermogenesis may be more pronounced with for example increasing BMI. If so, other mechanisms may overrule these effects among normal weight and underweight individuals. This is supported by a physiological study where high and low protein diets were fed to young, healthy, lean subjects; no differences were seen in insulin levels, appetite or total energy expenditure [19]. The potential negative energy balance with a high protein intake may be counteracted by a functional energy balance regulation. Other mechanisms of a high protein diet may lead to weight gain. A recent study [20] suggests that the weight gain associated with high protein intake in a broad population-based study cannot solely be ascribed to an anabolic effect on fat-free mass; greater protein intake was associated with gain in both fat-free mass and fat mass. Hence, high intake of protein may also stimulate growth of fat mass, possibly through interplay with insulin like growth factor-1 as seen in infants [21].

In the present study, the strongest association was found when matching on BMI and WC, simultaneously. The combination of these variables captures both total adiposity and body fat distribution. Body fat distribution is a better indicator of the adverse state of obesity than adiposity itself, as demonstrated in relation to mortality in the DCH cohort [22], [23]. The possibility that a beneficial effect of a high protein intake on weight control is more pronounced among individuals in an adverse state of obesity, with a combination of higher BMI and WC, needs to be investigated. Vergnaud *et al*. [7] found a significant interaction between BMI (below 25, 25–30 or above 30) and protein intake in relation to weight change. An association between greater protein intake and weight gain was seen in all three groups, but the strongest among individuals with BMI 25–30. However, interactions with other aspects of adiposity and body fat distribution were not investigated.

The literature on modifying observational data is growing. Although not related to dietary protein and weight, previous studies have mimicked a trial in observational data [13], [24]. Here other aspects were important to mimic, e.g. a wash-out period before initiation of a drug. Several studies have mimicked hypothetical interventions [25]–[32]. In relation to nutritional research Lajous et al. [32] investigated the association between change in fish intake and subsequent long-term risk of coronary heart disease by mimicking a hypothetical intervention of fish intake.

The method applied in the present study has the presumed advantage of mimicking the variation in exposure level followed by the intention-to-treat type of analysis of the trial. Further, it was possible to analyze the selected cohort participants according to the randomly assigned exposure of the trial participant. Otherwise, it may be problematic to compare results from an “as-treated” analysis in observational data with an intention-to-treat analysis in trial data. The intention-to-treat analysis does not necessarily reflect the actual exposure [33]. As seen in Figure 3 and Table 1, great variation of protein intake existed within the high and low protein groups of the trial. This shows that the trial did not achieve a clear distinction of exposure level in accordance with the randomization status. When matching participants from an observational cohort study with trial participants, as done in the present study, it was possible to select a subgroup similar to the trial participants including the variation in exposure level.

Matching was based on the Euclidean distance metric, but other methods could also have been used. For example the Mahalanobis distance [17], [18], [34] or on related extensions by propensity-like scores [35]. Future studies may explore such methods. Matching can be performed both with and, as done here, without replacement. An advantage of matching with replacement is that the match will not depend on initial sorting order of trial participants and that the distances will be globally minimized (given the used distance metric). However, some individuals, showing extreme values, may potentially end up with an unduly large influence on the results as a consequence of being selected multiple times.

From inspection of scree plots, four iterations were chosen, which may be considered as arbitrary, but it is unlikely that notable differences in results would be obtained by choosing, for instance, three or five iterations.

Several aspects of the trial participants could not be mimicked in the observational data. Despite 773 trial participants being randomized to the intervention, only 555 had information on dietary intake and, of these, 460 had information about FMI. However, the re-analysis of trial participants with data on diet and weight change between randomization and post-intervention (N = 441) showed results similar to the analyses of the initial trial. Inadequate matching on some variables was also a problem; the highest values of protein intake among trial participants could not get a good match in the observational data. This is probably because the highest intake in the trial generally goes beyond habitual intake reflected in observational data. However, the re-analysis of the DiOGenes trial data restricted to participants with protein intake below 30 E% showed a result essentially similar to the result of the original DiOGenes trial. Thus, these differences seemed not to influence the present study.

Various differences were present across the trial and the observational data, which can potentially be important for the results. These are discussed in the appendix note in file S1, and include differences in measurement methods, exposure, follow-up time as well as the differences between weight change and weight loss maintenance. However, the hypothesized beneficial effect of a high-protein diet on weight control may be assumed to be unaffected by these differences, which is supported by the results of the present study.

In conclusion, differences between the RCT and observational data were minimized wherever it seemed possible including dietary intake, participant characteristics and statistical analysis. This lead to a modified observational study where a better weight maintenance was seen in the high protein group than in the low protein group. The results suggest that participant selection and analytical strategy may be responsible for the conflicting results from observational studies and RCTs. Presence of overweight or obesity, and especially abdominal obesity, may be important to get a beneficial effect on weight maintenance of a high intake of protein. If so, the physiological mechanisms of protein intake in relation to weight control should be reconsidered. RCTs have found better weight control with high protein diets among overweight and obese individuals during 6–12 months, but there may be no obvious basis for recommending a high protein intake to normal weight individuals as a tool to better weight control. However, the present investigation and its contribution should be seen as explorative.

## Supporting Information

File S1 Contains the following supporting information files: **Appendix note:** Additional Discussion of differences between the trial and the cohort study. **Table S1:** Baseline characteristics of the modified DCH cohort data when different match-combinations are used. **Table S2:** Fully adjusted model including adjustment for total energy intake. Average annual weight change (kg/year) of cohort individuals matching trial participants randomized to high protein diet compared to cohort individuals matching trial participants randomized to low protein diet. **Table S3:** Results reported in the initial trial and results of supplementary analyses. **Figure S1–S4:** Scatter plots of matching performance.
(DOCX)
