# Supplementary material for: Body Characteristics, Dietary Protein and Body Weight Regulation. Reconciling Conflicting Results from Intervention and Observational Studies?
Source: PLoS One. 2014 Jul 3;9(7):e101134. doi: 10.1371/journal.pone.0101134 (PMC4081118; doi:10.1371/journal.pone.0101134)
Supplement: File S1 — Contains the following supporting information files: Appendix note: Additional Discussion of differences between the trial and the cohort study. Table S1: Baseline characteristics of the modified DCH cohort data when different match-combinations are used. Table S2: Fully adjusted model including adjustment for total energy intake. Average annual weight change (kg/year) of cohort individuals matching trial participants randomized to high protein diet compared to cohort individuals matching trial participants randomized to low protein diet. Table S3: Results reported in the initial trial and results of supplementary analyses. Figure S1–S4: Scatter plots of matching performance. (DOCX) [file pone.0101134.s001.docx]

**Supplementary material: file S1**

Body composition, dietary protein and body weight regulation. Reconciling conflicting results from intervention and observational studies?

Mikkel Z. Ankarfeldt, Lars Ängquist, Tanja Stocks, Marianne U. Jakobsen, Kim Overvad, Jytte Halkjær, Wim H.M. Saris, Arne Astrup, Thorkild I.A. Sørensen.

**Content:**

- **Appendix note:** Additional Discussion of differences between the trial and the cohort study.
- **Supplementary Table S1**: Baseline characteristics of the modified DCH cohort data when different match-combinations are used
- **Supplementary Table S2:** Fully adjusted model including adjustment for total energy intake. Average annual weight change (kg/year) of cohort individuals matching trial participants randomized to high protein diet compared to cohort individuals matching trial participants randomized to low protein diet
- **Supplementary Table S3**: Results reported in the initial trial and results of supplementary analyses.
- **Supplementary Figure S1-S4:** Scatter plots of matching performance

**Appendix note:** Additional Discussion of differences between the trial and the cohort study.

*Measurements:* The measurement methods differed in the two studies. For example, food intake was measured by a food record in the trial and by a food frequency questionnaire in the observational study, potentially affecting the matching quality.

*Context of exposure/diet:* The dietary exposure of the cohort data reflects habitual diet, while the trial participants are assigned fixed purpose-specific diets not being habitual. The differences between the ‘current user design’ of cohort data, and the ‘new-user design’ of trial data have previously been discussed in drug epidemiology [1], but may as well be relevant in the context of nutritional and in the present example. Bosse and Dixon [2] finds that beside an inter-individual spread of protein intake in the study population, also an intra-individual change in protein intake were important for studies to show an effect on weight change.

*Context of participation:* Trial participants are aware of participating in an intervention [3], which is not possible to reflect in observational data. E.g. participants in the trial had probably intention to maintain their weight loss, while the weight control intentions of the participants in the cohort data are not known.

*Follow-up:* The length of the follow-up period was different over the two studies, with five years in the observational Diet, Cancer and Health cohort, but just six months in the trial.

*Context of weight change:* The context of weight change differs in investigated trial and observational study, where an initial weight loss phase preceded the weight maintenance in the trial.

To gain further insights, the importance of the above-mentioned differences for the conflicting results may be considered in future studies.

**References**

1. Johnson ES, Bartman BA, Briesacher BA, Fleming NS, Gerhard T, et al. (2013) The incident user design in comparative effectiveness research. Pharmacoepidemiol.Drug Saf 22(1):1-6

2. Bosse JD, Dixon BM (2012) Dietary protein in weight management: a review proposing protein spread and change theories. Nutr.Metab (Lond) 9(1):81

3. Kaptchuk TJ (2001) The double-blind, randomized, placebo-controlled trial: Gold standard or golden calf? J Clin Epidemiol 54(6):541-549

**Supplementary Table S1**: Baseline characteristics of the modified DCH cohort data when different match-combinations are used^a^

|  | **Matching variables** | | | | | | | |
| --- | --- | --- | --- | --- | --- | --- | --- | --- |
|  | **Diet** | | **Diet and BMI** | | **Diet and WC** | | **Diet and FMI** | |
|  | N= 2 220 | | N= 2 220 | | N= 2 180 | | N=1 840 | |
|  | *Low protein*^b^ | *High protein* | *Low protein*^b^ | *High protein*^c^ | *Low protein*^d^ | *High protein*^e^ | *Low protein* | *High protein* |
|  | *N= 800* | *N=944* | *N= 800* | *N= 944* | *N= 784* | *N=928* | *N= 676* | *N=768* |
|  | *P50 (P5; P95)* | *P50 (P5; P95)* | *P50 (P5; P95)* | *P50 (P5; P95)* | *P50 (P5; P95)* | *P50 (P5; P95)* | *P50 (P5; P95)* | *P50 (P5; P95)* |
| *Protein E%* | 16.7 (12.3; 24.6) | 20.8 (14.8; 25.4) | 17.1 (12.5; 24.5) | 20.7 (14.9; 25.4) | 16.8 (12.4; 24.5) | 20.6 (15.0; 25.5) | 16.8 (12.4; 24.5) | 20.6 (14.9; 25.8) |
| *Carbohydrate E%* | 52.1 (31.3; 61.7) | 44.7 (31.5; 55.2) | 50.9 (31.5; 61.7) | 44.3 (32.9; 54.4) | 51.0 (32.0; 61.7) | 44.4 (32.1; 55.0) | 51.2 (31.8; 61.5) | 44.4 (31.6; 54.9) |
| *Fat E%* | 28.4 (19.3; 39.3) | 30.8 (22.8; 41.7) | 28.9 (19.5; 38.7) | 31.5 (23.5; 41.4) | 29.1 (19.5; 39.0) | 31.4 (23.3; 40.6) | 28.6 (19.5; 39.3) | 31.4 (23.1; 40.9) |
| *Alcohol E%* | 2.3 (0.0; 11.5) | 2.8 (0.1; 14.7) | 2.3 (0.1; 12.8) | 2.6 (0.1; 15.8) | 2.1 (0.1; 11.7) | 2.5 (0.1: 15.3) | 2.2 (0.1; 11.8) | 2.7 (0.1; 15.8) |
| *Glycemic index* | 58.9 (50.3; 67.1) | 58.9 (50.3; 68.1) | 59.0 (50.7; 66.9) | 58.8 (51.0; 67.8) | 58.9 (50.9; 66.9) | 58.9 (50.8; 67.8) | 59.1 (51.3; 66.9) | 58.8 (50.6; 68.0) |
| *Energy (MJ)* | 8.1 (4.8; 13.1) | 7.9 (4.8; 12.5) | 8.1 (5.0; 13.1) | 7.9 (4.9; 12.4) | 8.2 (4.8; 13.1) | 8.0 (4.9; 12.4) | 8.1 (4.8; 13.0) | 8.0 (4.9; 12.5) |
| *Weight (kg)* | 72.0 (54.5; 98.3) | 74.2 (56.1; 101.9) | 80.5 (62.8; 109.4) | 83.1 (64.1; 112.0) | 80.0 (63.3; 111.0) | 81.5 (62.6; 109.1) | 81.5 (64.4; 110.1) | 82.9 (64.5; 113.0) |
| *BMI (kg/m^2^)* | 25.1 (20.2; 33.1) | 25.7 (20.7; 33.3) | 28.6 (23.3; 38.1) | 29.1 (23.8; 37.4) | 28.0 (22.6; 38.0) | 28.0 (22.8; 37.3) | 28.7 (23.3; 38.3) | 28.9 (23.3; 38.0) |
| *WC (cm)* | 84.0 (69.0; 108.0) | 87.0 (69.0; 110.0) | 92.5 (74.0; 116.0) | 94.0 (77.0; 117.0) | 93.0 (78.0; 117.0) | 94.0 (78.0; 116.0) | 94.0 (79.0; 117.0) | 95.0 (78.0; 116.0) |
| *FMI* | 7.8 (4.0; 13.9) | 8.0 (4.5; 13.9) | 10.3 (5.8; 17.4) | 10.6 (5.8; 17.7) | 10.0 (5.3; 17.5) | 10.0 (5.3; 17.4) | 10.5 (5.6; 17.7) | 10.4 (5.6; 17.9) |
| *Age (years)* | 53 (50; 58) | 53 (50; 58) | 53 (50; 58) | 53 (50; 58) | 54 (50; 58) | 53 (50; 58) | 54 (50; 58) | 53 (50; 58) |
| *Weight change (kg)*^f^ | -0.02 (-1.29; 1.41) | 0.00 (-1.46; 1.47) | -0.06 (-1.66; 1.60) | -0.11 (-2.05; 1.50) | -0.06 (-1.67; 1.73) | -0.06 (-1.90; 1.37) | -0.04 (-1.72; 1.73) | -0.07 (-2.07; 1.55) |

Abbreviations: BMI, body mass index; DCH, Diet Cancer and Health; E %, percent of energy intake; FMI, fat mass index; P50, median; P5, 5^th^ percentile; P95, 95^th^ percentile; WC, waist circumference.

^a^Diet: Protein E%, carbohydrate E%, glycemic index. Median, 5^th^ and 95^th^ percentile of baseline variables and weight change. High and low protein groups in modified cohort data is based on the randomization status of the matched trial participants. Information on control groups is not shown. Mean values across matches of the ten random orders of the trial dataset.

^b^Information on FMI available in N=798

^c^Information on FMI available in N=942

^d^Information on FMI available in N=782

^e^Information on FMI available in N=925

^f^Average annual change in weight from baseline to follow-up (mean 5.3 years).

**Supplementary Table S2:** Fully adjusted model including adjustment for total energy intake. Annual weight change effect when comparing cohort individuals matching trial participants randomized to either high or low protein intake^a^

| **Matching variables** | **Fully adjusted model including energy intake**^b^ | |
| --- | --- | --- |
|  | *β (range)* | *p-value*^c^ *(range)* |
| *Diet* | -0.001 (-0.023; 0.023) | 0.992 (0.590; 0.983) |
| *Diet and BMI* | -0.089 (-0.150; -0.048) | 0.087 (0.004; 0.360) |
| *Diet and WC* | -0.086 (-0.128; -0.029) | 0.088 (0.012; 0.565) |
| *Diet and FMI* | -0.078 (-0.130; -0.039) | 0.173 (0.023; 0.498) |
| *Diet, WC and BMI* | -0.141 (-0.179; -0.077) | 0.008 (<0.001; 0.151) |

Abbreviations: BMI, body mass index; E%, percent of energy intake; FMI, fat mass index; WC, waist circumference.

^a^Five match-combinations: Diet only (Protein E%, carbohydrate E% and glycemic index), diet in combination with BMI, WC or FMI, or diet, BMI and WC. Multiple linear regression analysis was used. Exposure was indicator variables (yes/no) of matched randomization groups: high protein, high glycemic index, control. Outcome was average annual weight change between baseline and follow-up (mean 5.3 years). β = difference in weight change between high and low protein group. β and p-values presented as means and summary statistics, respectively, complemented with corresponding ranges across matches of the ten random orders of the trial dataset.

^b^Adjustment for sex (male/female without hormone use/female with hormone use), baseline BMI, age, physical activity (4 groups: inactive, moderately inactive, moderately active, active), education (4 groups: primary school, technical/professional school, secondary school, university degree) and intake of fibers (g/day) and alcohol (E%).

^c^Summary p-values, derived from the means of the β-estimates and of the corresponding standard errors, respectively, over the ten individual matches

**Supplementary Table S3**: Results reported in the initial trial and results of supplementary analyses.

| **Results reported in the initial DiOGenes trial**^a^ | | | |
| --- | --- | --- | --- |
|  | *N* | *β (95 %CI)* | *p* |
| *Intention-to-treat analysis* | 773 | -0.93 (-1.55; -0.31) | 0.003 |
| *Completion analysis* | 548 | -1.44 (-2.33; -0.50) | 0.020 |
| **Supplementary analyses of the DiOGenes trial**^b^ | | | |
|  | *N* | *β (95 %CI)* | *p* |
| *Participants with dietary data* | 440 | -1.20 (-2.35; -0.05) | 0.041 |
| *Participants with dietary data and protein E% <30* | 420 | -1.31 (-2.49; -0.13) | 0.030 |

^a^Analyzed according to randomization status. Intention-to-treat analysis: All randomized participants; missing data were imputed. Completion analysis: Participants with available data from randomization and follow-up. Found in Larsen *et al.* N Engl J Med. 2010;363(22):2102-13

^b^Analyzed according to randomization status. Outcome was weight change between randomization and end of intervention. Adjusted for body-mass index (continuous), sex (female/male). Multiple linear regression analysis. β = adjusted mean difference of weight between high and low protein group.

**Supplementary Figure S1-S4:** Scatter plots of matching performance

**Supplementary Figure S1***:* Matching performance. Scatter plot of modified cohort participants vs. the corresponding, matched trial participants. Matching based on protein E%, carbohydrate E% and glycemic index. Four participants from the cohort data matched with every trial participants. Mean values across matches of the ten random orders of the trial dataset are presented. The line of equality, y=x, indicates a perfect match. Red square marker: low protein group. Blue triangle marker: high protein group. Control group is not shown. Trial participants N=555, defined by having available measurements of diet and BMI; matched modified cohort participants N=2 220. E%: percent of energy intake

**Supplementary Figure S2:** Matching performance. Scatter plot of modified cohort participants vs. the corresponding, matched trial participants. Matching based on protein E%, carbohydrate E%, glycemic index and BMI. Four participants from the cohort data matched with every trial participants. Mean values across matches of the ten random orders of the trial dataset are presented. The line of equality, y=x, indicates a perfect match. Red square marker: low protein group. Blue triangle marker: high protein group. Control group is not shown. Trial participants N=555, defined by having available measurements of diet and BMI; matched modified cohort participants N=2 220. BMI: body mass index (kg/m^2^). E%: percent of energy intake

**Supplementary Figure S3**: Matching performance. Scatter plot of modified cohort participants vs. the corresponding, matched trial participants. Matching based on protein E%, carbohydrate E%, glycemic index and WC. Four participants from the cohort data matched with every trial participants. Mean values across matches of the ten random orders of the trial dataset are presented. The line of equality, y=x, indicates a perfect match. Red square marker: low protein group. Blue triangle marker: high protein group. Control group is not shown. Trial participants N=545, defined by having available measurements of diet and WC; matched modified cohort participants N=2 180. E%: percent of energy intake. WC: waist circumference (cm).

**Supplementary Figure S4**: Matching performance. Scatter plot of modified cohort participants vs. the corresponding, matched trial participants. Matching based on protein E%, carbohydrate E%, glycemic index and FMI. Four participants from the cohort data matched with every trial participants. Mean values across matches of the ten random orders of the trial dataset are presented. The line of equality, y=x, indicates a perfect match. Red square marker: low protein group. Blue triangle marker: high protein group. Control group is not shown. Trial participants N=460, defined by having available measurements of diet and FMI; matched modified cohort participants N=1 840. FMI: Fatmass index. E%: percent of energy intake.
